# Optogenetic and Endogenous Modulation of Ca^2+^ Signaling in Schwann Cells: Implications for Autocrine and Paracrine Neurotrophic Regulation

**DOI:** 10.3390/ijms26189082

**Published:** 2025-09-18

**Authors:** Tomohiro Numata, Moe Tsutsumi, Kaori Sato-Numata

**Affiliations:** 1 Department of Integrative Physiology, Graduate School of Medicine, Akita University, Akita 010-8543, Japan; satokao@med.akita-u.ac.jp; 2 MIRAI Technology Institute, Shiseido Co., Ltd., Yokohama 220-0011, Japan; moe.tsutsumi@shiseido.com

**Keywords:** peripheral nerve regeneration, calcium signaling, Schwann cells, optogenetics, secretome

## Abstract

Schwann cells (SCs) are central players in peripheral nerve repair, facilitating axonal regrowth, remyelination, and modulation of the regenerative microenvironment. A pivotal driver of these functions is intracellular Ca^2+^ signaling, regulated by both endogenous Ca^2+^-permeable ion channels and engineered optogenetic actuators. Recent developments in optogenetics, particularly the application of Ca^2+^-permeable channelrhodopsins such as CapChR2, have enabled precise, light-controlled activation of SCs, allowing for targeted investigation of Ca^2+^-dependent pathways in non-neuronal cells. This review synthesizes emerging evidence demonstrating that optogenetically or endogenously induced Ca^2+^ influx in SCs leads to the release of a diverse set of neurotrophic and regulatory factors. These Ca^2+^-triggered secretomes modulate SC phenotypes and surrounding neurons, orchestrating axon regeneration and myelin repair via autocrine and paracrine mechanisms. We further discuss the roles of key endogenous Ca^2+^ channels—including transient receptor potential (TRP) channels and store-operated Ca^2+^ entry (SOCE; STIM/Orai)—in orchestrating SC activation under physiological and injury-induced conditions. By integrating insights from optogenetic manipulation and intrinsic signaling biology, this review proposes a conceptual framework in which Ca^2+^-triggered SC secretomes act as structural and functional scaffolds for nerve repair. We highlight how SC-derived factors shape the regenerative niche, influence adjacent neurons and glia, and modulate repair processes in peripheral and autonomic nerves.

## 1. Introduction

Peripheral nerve injuries represent a considerable clinical and socioeconomic burden. Epidemiological data indicate an incidence of 13–23 per 100,000 persons annually worldwide [1], with a nationwide Swedish study reporting a decline from 18.6 to 12.9 per 100,000 between 2008 and 2022 (men: 23.1 → 15.6; women: 14.1 → 10.1 per 100,000) [2]. In Germany, the direct acute treatment costs per case ranged from €3418 to €5360, with rehabilitation and productivity losses raising the total per-patient socioeconomic burden to over €17,600; approximately 30% of affected individuals received lifetime disability pensions averaging €102,167 [3]. Despite this considerable burden, recovery is often incomplete due to insufficient axonal regeneration and remyelination [4,5]. In response to such injury, Schwann cells (SCs) undergo a remarkable phenotypic reprogramming into a “repair” state, characterized by dedifferentiation, downregulation of myelin genes, and upregulation of regeneration-associated transcriptional programs. These repair SCs organize into Bands of Büngner—longitudinally aligned cellular structures that provide both physical and molecular guidance cues for regenerating axons [5,6].

SCs contribute to peripheral nerve regeneration by secreting neurotrophic factors and extracellular matrix (ECM) components, supporting both neuronal survival and axonal guidance [5,6,7]. Beyond acute secretory outputs, patterned Ca^2+^ signals are poised to drive SC gene-reprogramming during the repair switch by engaging Ca^2+^-sensitive pathways (e.g., calcineurin/NFAT, ERK/c-Jun, PI3K/AKT) and myelin-regulatory transcription factors such as MYRF, thereby coupling ionic signaling to transcriptional plasticity. These regenerative functions are tightly regulated by intracellular Ca^2+^ signaling, which governs a range of SC activities including proliferation, mitochondrial dynamics, phenotypic plasticity, and neuro-supportive interactions [8,9,10].

Multiple Ca^2+^-permeable ion channels orchestrate these processes by mediating Ca^2+^ influx in response to diverse physiological stimuli. Key Ca^2+^-permeable channels include TRP family members, mechanosensitive Piezo channels, P2X receptors, and store-operated Ca^2+^ entry (SOCE; STIM/Orai) components, all of which enable SCs to respond dynamically to microenvironmental cues [11,12,13,14,15,16]. Mechanosensitive channels like Piezo2 also contribute to SC responsiveness by converting mechanical stimuli into Ca^2+^ signals that influence secretory and supportive functions [17], while recent comprehensive analyses have identified PIEZO1 and PIEZO2 as among the most abundant mechanosensitive channels in SCs [18]. In addition, SOCE, including STIM1/2 and Orai1–3, with TRPC1 potentially contributing in a context-dependent manner, form a cooperative signaling complex that maintains Ca^2+^ homeostasis in response to endoplasmic reticulum Ca^2+^ depletion and other physiological demands [19]. Classical voltage-gated Ca^2+^ channels (VGCCs) are also functionally expressed in cultured mouse SCs, especially under conditions of neuron–glia interaction and elevated intracellular cAMP levels, suggesting an activity-dependent regulatory mechanism [20].

**Figure 1:**
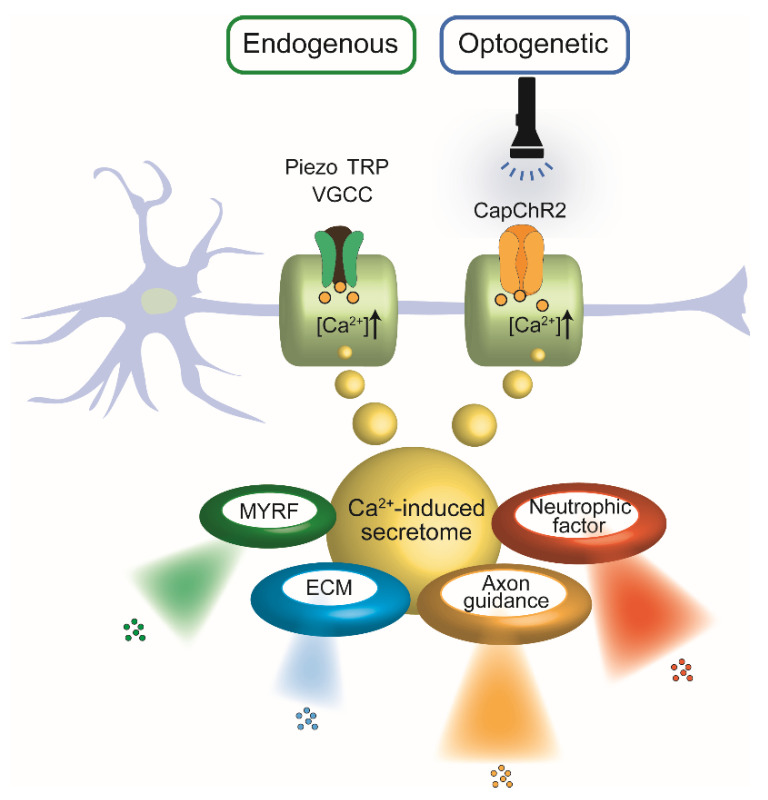
Shared Ca^2+^-dependent secretory pathways in SCs activated by endogenous or optogenetic stimuli. TRP: transient re-ceptor potential; Piezo: mechanosensitive Piezo channels; VGCC: voltage-gated ion channels; CapChR2: a light-sensitive Ca^2+^-permeable channelrhodopsin; MYRF: myelin regulatory factor; ECM: extracellular matrix components.

In parallel, optogenetic tools have emerged as precise actuators of intracellular Ca^2+^ signals. Channelrhodopsin variants such as CapChR2, engineered for enhanced Ca^2+^ permeability, enable light-controlled modulation of SC behavior with high spatial and temporal resolution [21,22,23]. These tools enable selective activation of SCs in co-culture or in vivo settings, facilitating the analysis of Ca^2+^-dependent processes such as neurotrophic factor release, cytoskeletal reorganization, and regenerative program induction. As illustrated in Figure 1, both endogenous and optogenetic activation routes converge on Ca^2+^-mediated signaling in SCs, yielding common downstream outcomes including neurotrophic factor secretion and regenerative remodeling.

Schematic representation illustrating the convergence of endogenous and optogenetic Ca^2+^ entry pathways in SCs and their shared downstream outputs. Left: Endogenous Ca^2+^ influx occurs via mechanosensitive and voltage-gated ion channels, including Piezo, TRP, and VGCCs, in response to physical or chemical stimuli. Right: Optogenetic activation of CapChR2, a light-sensitive Ca^2+^-permeable channelrhodopsin, enables precise temporal control of intracellular Ca^2+^ levels via light exposure. In both pathways, elevated intracellular Ca^2+^ triggers a common secretome, comprising: Neurotrophic factors, Axon guidance cues, Extracellular matrix (ECM) components, Myelin regulatory factor (MYRF).

This review synthesizes current knowledge on the molecular architecture and functional implications of Ca^2+^ signaling in SCs, with a particular emphasis on the interface between intracellular Ca^2+^ dynamics and the secretory phenotype. By integrating findings from both optogenetic and physiological activation paradigms, we explore how Ca^2+^-triggered SC-derived secretomes influence the surrounding neural microenvironment and coordinate axon-glia interactions essential for peripheral nerve regeneration.

## 2. Endogenous Ca^2+^-Permeable Channels in SCs

Among the multiple signaling inputs governing SC plasticity, Ca^2+^ influx emerges as a versatile regulator that coordinates their injury response, metabolic adaptation, and trophic support. A variety of endogenous Ca^2+^-permeable ion channels are expressed in SCs, and these channels respond to electrical, mechanical, osmotic, and chemical stimuli to shape intracellular signaling dynamics. In this section, we summarize the major classes of Ca^2+^-permeable channels in SCs, focusing on their molecular identities, physiological functions, and specific contributions to SC plasticity.

**Figure 2:**
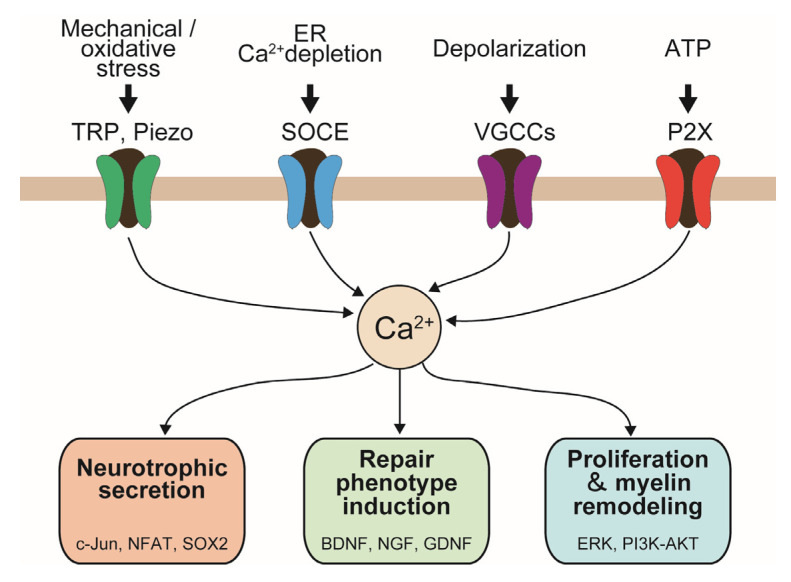
Association of endogenous Ca^2+^-permeable channels with functional outcomes in SCs. TRP: transient re-ceptor potential; Piezo: mechanosensitive Piezo channels; SOCE: store-operated Ca^2+^ entry; VGCC: voltage-gated ion channels; P2X: a family of ligand-gated ion channels; ATP: adenosine triphosphate. NFAT: nuclear factor of activated T cells; BDNF: brain-derived neurotrophic factor; NGF: nerve growth factor; GDNF: glial cell line-derived neurotrophic factor; ERK: extracellular signal-regulated kinase; PI3K-AKT: phosphatidylinositol 3-kinase/Serine-Threonine kinase.

The major Ca^2+^-permeable channels expressed in SCs, their representative stimuli, and their predominant functional outcomes are summarized in Figure 2. This schematic highlight how distinct channels—including TRP, Piezo, STIM/Orai, VGCCs, and P2X receptors—mediate extracellular Ca^2+^ influx and contribute to repair phenotype induction, neurotrophic secretion, and proliferation/myelin remodeling, often with overlapping functions.

TRPV4, TRPA1, and TRPM7 mediate Ca^2+^ influx in response to osmotic, mechanical, and oxidative stress, supporting the induction of a repair phenotype and contributing to myelin remodeling. Piezo channels decode mechanical stretch to regulate neurotrophic factor secretion and myelination. STIM/Orai (SOCE ± TRPC1) respond to ER Ca^2+^ depletion and mediate both neurotrophic factor secretion and proliferation. VGCCs, activated by depolarization, contribute not only to NGF secretion but also to proliferation and myelin remodeling. P2X receptors, activated by ATP, regulate cytokine and BDNF release, thereby promoting proliferation and remyelination. Extracellular Ca^2+^ influx may be mediated by single or multiple channel types, with overlapping contributions to distinct functional outcomes.

### 2.1. Voltage-Gated Ca^2+^ Channels in SCs

VGCCs are integral membrane proteins that mediate Ca^2+^ influx in response to depolarizing stimuli. Structurally, these channels are hetero-oligomeric complexes composed of a central pore-forming α_1_ subunit—encoded by CACNA1 genes—and auxiliary β, α_2_δ, and γ subunits, which modulate trafficking, gating, and pharmacological properties [24,25]. Based on their activation thresholds and pharmacological profiles, VGCCs are classified into high-voltage-activated (HVA) subtypes—L-type, P/Q-type, N-type, and R-type—and low-voltage-activated (LVA) T-type channels [24,25].

In SCs, both T-type–like and L-type–like VGCC currents have been identified across several experimental models, including murine dorsal root ganglion (DRG) co-cultures [20,26], rat peripheral nerves [27], and the glia of the squid giant axon (comparative model) [28]. In mouse SC cultures, T-type currents exhibit transient activation at low thresholds and are insensitive to L-type modulators such as nifedipine and Bay K8644, whereas L-type currents activate at higher thresholds, inactivate slowly, and are sensitive to these agents [20].

Notably, VGCC expression and activity in SCs are dynamically modulated by neuron-derived factors and intracellular cAMP signaling. Co-culture with DRG neurons enhances VGCC expression in SCs, while their removal markedly reduces detectable Ca^2+^ currents. This downregulation can be prevented or reversed by the application of cAMP analogs—including CPT-cAMP, db-cAMP, or forskolin—indicating that neuron–glia communication through diffusible signals and cAMP-dependent pathways plays a key role in maintaining VGCC expression [26].

VGCCs, though less studied than other Ca^2+^ channels, nonetheless mediate activity-dependent responses in SCs. Their regulation by neuronal cues positions them as potential mediators of context-specific glial adaptation, especially in environments where electrical activity or second-messenger signaling is altered, such as during injury or repair.

Functionally, VGCCs in SCs contribute to neurotrophic factor release; in cultured SCs, electrical stimulation enhances NGF secretion via T-type channel–mediated Ca^2+^ influx with additional mobilization from IP_3_- and ryanodine-sensitive stores [27]. These findings reinforce the role of VGCCs as activity-dependent regulators in neuron–glia communication. Similarly, in the squid model, L-type VGCCs contribute to membrane potential regulation and are sensitive to nifedipine, underscoring the evolutionary conservation of Ca^2+^ signaling mechanisms in glial physiology [28].

Collectively, these findings indicate that SCs, although non-excitable, possess functionally relevant VGCCs that allow them to sense and respond to electrical and biochemical cues in the peripheral nerve environment. This Ca^2+^ influx system is particularly significant in developmental or injury contexts, where SCs transition into repair phenotypes and orchestrate axon regeneration and remyelination.

### 2.2. TRP Channels

TRP channels are a superfamily of non-selective cation channels that mediate Ca^2+^ influx in response to diverse physical and chemical stimuli, including temperature, osmotic stress, reactive oxygen species (ROS), and lipid-derived messengers. Structurally, TRP channels assemble as tetrameric complexes, with each subunit containing six transmembrane domains (S1–S6) and a pore loop between S5 and S6. Based on sequence homology, they are classified into subfamilies such as TRPC (canonical), TRPV (vanilloid), TRPA (ankyrin), and TRPM (melastatin), several of which are functionally expressed in SCs [29,30].

Among them, members of the TRP channel family—particularly TRPA1, TRPV4, and TRPM7—play essential roles in transducing oxidative, mechanical, and inflammatory signals into Ca^2+^ responses under both physiological and pathological conditions in SCs.

TRPA1 is activated by ROS and inflammatory mediators and is upregulated in SCs after nerve injury. Its activation promotes NOX1-dependent H_2_O_2_ production, sustaining local oxidative stress and macrophage infiltration, thereby linking redox signaling to Ca^2+^ influx during neuroinflammation [11]. TRPV4, a warm temperature-sensitive channel, is also upregulated in SCs after peripheral nerve injury. It contributes to demyelination by mediating Ca^2+^ entry in response to TRPV4 activator, GSK1016790A, and genetic or pharmacological inhibition of TRPV4 mitigates demyelination and improves remyelination and functional recovery [12]. TRPM7 is a unique, ubiquitously expressed ion channel with an attached kinase domain, and it is essential for cellular and organismal survival [31], regulates Ca^2+^-dependent volume homeostasis and is involved in Wallerian degeneration. Pharmacological inhibition of TRPM7 protects against axonal and myelin breakdown, highlighting its involvement in Ca^2+^-dependent degenerative processes [32].

These TRP channels share the capacity to mediate stimulus-specific Ca^2+^ influx, enabling SCs to flexibly respond to diverse microenvironmental challenges such as oxidative stress, mechanical deformation, and inflammatory injury. Their coordinated activity supports key functions including inflammatory modulation, cytoskeletal remodeling, and regulation of the regenerative phenotype.

Complementing TRP-mediated Ca^2+^ entry, store-operated Ca^2+^ entry (SOCE) is another crucial pathway in SCs. Upon endoplasmic reticulum (ER) Ca^2+^ depletion, the ER-resident sensor STIM1 translocates to the plasma membrane and activates Orai1 channels. TRPC1, a canonical TRP channel, contributes to this complex, forming a parallel or supportive Ca^2+^-conducting pathway [33]. Notably, PMP22, a myelin-specific protein implicated in Charcot-Marie-Tooth disease, interacts with STIM1 and modulates TRPC1-dependent SOCE activity, suggesting a direct link between Ca^2+^ homeostasis and demyelinating pathologies [19]. In support of this connection, mutations in TRPV4—another TRP channel expressed in SCs—have been shown to cause Charcot-Marie-Tooth disease type 2C, indicating that dysregulation of TRP-mediated Ca^2+^ signaling can directly contribute to peripheral neuropathies [34].

Together, TRP channels and SOCE channels allow SCs to maintain plastic Ca^2+^ signaling in both steady-state and regenerative contexts. These mechanisms operate independently of membrane depolarization, offering versatile means to modulate SC behavior in response to extracellular stimuli—particularly during peripheral nerve repair.

### 2.3. Piezo Channels

Piezo channels are a class of mechanically activated ion channels that open in response to membrane tension or deformation. Among them, Piezo1 and Piezo2 are the two principal isoforms identified in mammals [35]. These channels assemble as homotrimeric structures, forming propeller-like transmembrane complexes with 114 membrane-spanning segments (3 × 38 per trimer), which function as exquisitely sensitive mechanotransducers that translate physical membrane distortion into non-selective cation influx, including Ca^2+^ [36,37,38]. Piezo1, initially recognized in endothelial cells, has been implicated as a key mediator of shear stress responses [39,40], whereas Piezo2 plays a dominant role in mechanosensory neurons and glial cells.

In SCs, Piezo2 has recently been identified as a critical regulator of mechanosensitive Ca^2+^ signaling. Suttinont et al. (2024) indicated that Piezo2 is functionally expressed in SCs and contributes to cell volume regulation and the control of neurotrophic factor release [17]. Specifically, mechanical stimulation or swelling activates Piezo2, leading to Ca^2+^ influx that triggers downstream signaling events such as cytoskeletal reorganization and secretion of brain-derived neurotrophic factor (BDNF). Complementing these findings, Acheta et al. (2022) conducted a comprehensive analysis of mechanosensitive ion channels in SCs and identified Piezo1 and Piezo2 as among the most abundant [18]. Notably, they reported counterbalanced roles in myelination. Piezo1 transiently inhibited radial and longitudinal myelin development, whereas Piezo2 was required for proper myelin formation. Moreover, Piezo1 was shown to regulate YAP/TAZ activity, linking mechanotransduction to transcriptional programs governing SC development and regeneration. These observations point to a functional interplay between Piezo1 and Piezo2 in SC maturation and mechanosensory responsiveness—actions that are crucial for SC adaptation to injury-induced mechanical stress and for supporting axonal regeneration.

Consistent with this view, our recent findings [17], show that Piezo2 expression increases during phenotypic maturation of SCs, accompanied by enhanced Ca^2+^ responses to mechanical stimuli. These results suggest that Piezo2 functions as a context-sensitive mechanosensitive integrator whose activation becomes functionally relevant as SCs acquire a myelin-competent phenotype. In the injury setting, where SCs transition to a dedifferentiated, proliferative “repair SC” state distinct from myelinating SCs, Piezo2 could likewise help decode mechanical inputs from the extracellular matrix, axonal growth cones, or perineural edema during Wallerian degeneration and nerve regrowth. Supporting this role, Piezo2 activation has been shown to maintain RhoA activity, promote stress fiber formation, and enhance motility in other cell types such as metastatic cancer cells [41]. Furthermore, Piezo2-mediated Akt signaling has been implicated in transcriptional programs that facilitate phenotypic plasticity [42]. Thus, Piezo2 likely acts as a context-sensitive mediator of glial differentiation and mechanotransduction during peripheral nerve repair.

TRPM7, discussed in Section 2.2, also contributes to mechanical Ca^2+^ sensing and may act in parallel with Piezo2 during injury-induced stress. TRPM7, a stretch-sensitive channel with kinase activity [13,14,43], complements Piezo2 in mediating Ca^2+^ responses to mechanical stress. Its upregulation during Wallerian degeneration and role in limiting demyelination suggest overlapping functions with Piezo2 in mechanical signal transduction. Importantly, TRPM7 is upregulated during Wallerian degeneration, and its inhibition reduces demyelination and axonal degradation in peripheral nerve injury models [32]. Together, Piezo2 and TRPM7 may cooperate to decode mechanical changes in the microenvironment, enabling SCs to adapt their phenotype and promote regenerative outcomes after injury.

### 2.4. P2X Channels

Lastly, P2X receptors are a family of ligand-gated ion channels that are directly activated by extracellular adenosine triphosphate (ATP), mediating rapid influx of cations such as Na^+^, K^+^, and Ca^2+^ [44,45]. Structurally, they are trimeric assemblies composed of seven known subunits (P2X1–P2X7), each containing two transmembrane domains, intracellular N- and C-termini, and a large extracellular ATP-binding loop responsible for ligand recognition and channel gating [46].

Upon ATP release during injury, P2X7 receptors mediate Ca^2+^ influx and trigger downstream inflammatory and regenerative signaling, including cytokine secretion and modulation of SC migration and differentiation [47,48]. P2X7 receptors, characterized by their high activation threshold and the ability—under sustained ATP stimulation—to form large, non-selective pores that permit uptake of molecules in the ~600–900 Da range, play a context-dependent role in SC biology. Activation of P2X7 by potent agonists such as BzATP promotes Ca^2+^ influx that regulates cytokine release (e.g., TNF-α, IL-1β), cell volume changes, and expression of intercellular communication proteins such as connexins; note that BzATP is potent but not fully selective. Moreover, as regeneration progresses, P2X7 activation shifts SC behavior from early proliferative repair states toward migration and (re)myelinating differentiation, changes that support subsequent remyelination in vivo [49]. In vivo, pharmacological activation of P2X7 accelerates functional nerve recovery and myelin repair, whereas antagonism impairs these processes, positioning P2X7 as a promising therapeutic target for modulating SC fate and regeneration.

P2X4 receptors, on the other hand, are upregulated in SCs following peripheral nerve injury and are largely localized in intracellular compartments such as lysosomes, with translocation to the plasma membrane upon inflammatory stimulation [50]. When activated in SCs (distinct from microglia), P2X4 has been reported to enhance BDNF release, which in turn supports remyelination and motor/sensory recovery. In parallel, a miR-363-5p–P2X4 double-negative loop operates as follows: miR-363-5p restrains P2X4 during postnatal development but declines after injury, relieving P2X4 inhibition and facilitating SC dedifferentiation and migration [51].

Collectively, purinergic signaling through P2X receptors equips SCs with the ability to detect ATP as a damage-associated molecular pattern (DAMP) and convert it into a spectrum of regenerative responses. These responses include phenotype modulation, cytokine secretion, axon guidance, and remyelination. The dual roles of P2X7 and P2X4—balancing inflammation, differentiation, and neurotrophic support—underscore their importance as molecular switches in the dynamic SC response to peripheral nerve injury.

Taken together, endogenous Ca^2+^-permeable ion channels provide SCs with a versatile signaling platform to sense and respond to a wide range of environmental stimuli, including mechanical stress, ATP release, and osmotic changes. In the Wallerian degeneration milieu, axonal injury elevates axoplasmic Ca^2+^ and releases ATP and other DAMPs, which can secondarily drive SC Ca^2+^ entry via purinergic (P2X) receptors and sensitize TRP, Piezo, and SOCE pathways. Thus, degenerating axons likely act as indirect Ca^2+^ sources for neighboring SCs. Notably, the expression and function of these channels are tightly coupled to the differentiation state of SCs, implying that distinct channel repertoires may operate in immature, myelinating, and repair phenotypes. While Ca^2+^ pumps and exchangers such as PMCA and NCX mainly regulate extrusion or buffering, injury-evoked Ca^2+^ entry into SCs is more plausibly mediated by channels (P2X, TRP, Piezo, SOCE, VGCC). Reverse-mode NCX may contribute to depolarized niches, but evidence in SCs during Wallerian degeneration remains limited. Further studies are warranted to elucidate the full complement of Ca^2+^-permeable channels across SC subtypes and developmental stages, which may uncover new molecular targets for enhancing peripheral nerve repair.

## 3. Functional Implications of Ca^2+^ Signaling in Regenerating SCs

After peripheral nerve injury, SCs undergo a dynamic transformation into a repair phenotype, characterized by increased motility, upregulation of trophic factor expression, and the formation of Bands of Büngner that guide axonal regrowth [52,53,54]. This phenotypic switch is tightly regulated by various signaling cascades, among which Ca^2+^ signaling plays a central role.

Ca^2+^ influx through ion channels such as TRP, store-operated Ca^2+^ entry (SOCE), and mechanosensitive channels like Piezo2 may initiate key downstream pathways, including ERK, c-Jun [53,54,55], PI3K/AKT/PTEN [56], and, that promote cellular reprogramming and plasticity. Anatomical specializations of myelinating SCs—including Schmidt–Lanterman incisures (SLIs) and paranodal loops—are enriched with functional gap junctions, which serve as radial conduits enabling direct transfer of ions and small molecules between adjacent myelin layers [57]. These structures may thus function as internal “highways” for Ca^2+^ microdomain propagation, efficiently relaying periaxonal signals across the sheath and coordinating localized Ca^2+^ dynamics with myelin remodeling. As described above, Piezo2 activation links mechanotransduction to neurotrophic signaling [17], a function integral to the injury-adaptive transformation of SCs.

These pathways coordinate cytoskeletal remodeling, adhesion molecule regulation, and transcriptional reprogramming necessary for SC migration and the establishment of axon-guiding Bands of Büngner. Notably, SC plasticity underlies their ability to adapt to injury environments and support long-term regeneration, even in aged or chronically denervated tissues [58,59,60]. Although the involvement of Ca^2+^ signaling in epithelial-to-mesenchymal transition (EMT)-like changes and stemness-associated gene regulation in SCs remains to be fully elucidated, parallels have been suggested based on similar mechanisms described in cancer biology [61,62]. These findings suggest that sustained Ca^2+^ signaling can engage partial EMT(P-EMT)-like programs that promote SC plasticity during regeneration.

Recent bioinformatics analyses, including those assessing perineural invasion in cancer [63], suggest that prolonged or aberrant Ca^2+^ signaling may act as a key regulator of phenotypic transitions in cells with regenerative or invasive potential. Studies in oncology have demonstrated that sustained Ca^2+^ influx can trigger P-EMT programs, particularly through the internalization of E-cadherin and activation of the calmodulin–CaMKII signaling cascade, leading to enhanced cell migration and invasiveness [61,62]. Thus, Ca^2+^ functions as a master regulator of phenotypic reprogramming, with parallels to EMT in oncology. Section 4 further explores how optogenetic tools can precisely engage these pathways.

Although SCs operate in a distinct physiological context, the overlap in Ca^2+^-sensitive pathways regulating epithelial–mesenchymal plasticity suggests a conserved role for Ca^2+^ as a master regulator of cellular adaptability. In this regard, modulating Ca^2+^ signaling in SCs—via TRP channels, Piezo2, or optogenetic tools such as CapChR2—could represent a strategy to fine-tune their repair phenotype and neurotrophic function, with potential translational implications.

This secretory activity, driven by Ca^2+^ influx, is further explored in Section 5. For example, endothelial cell-derived exosomes enhance SC repair phenotypes via miR-199-5p and PI3K/AKT signaling [64], illustrating the broader network of Ca^2+^-linked intercellular communication in the regenerative microenvironment.

Collectively, these findings underscore the multifaceted role of Ca^2+^ signaling in orchestrating regenerative responses in SCs, spanning from phenotypic transformation and motility to mechanosensitive regulation of neurotrophic support. Targeting Ca^2+^-responsive pathways, particularly those involving Piezo2, may provide new therapeutic leverage for enhancing peripheral nerve regeneration. In this context, the customized design of conducting polymer architectures, such as PEDOT-based microstructures, offers promising opportunities to monitor and interface with electrochemical signals in damaged nerves [65,66]. By enabling simultaneous sensing and modulation of bioelectrical activity, these materials may provide powerful platforms to bridge Ca^2+^-dependent SCs dynamics with regenerative bioelectronics.

## 4. Optogenetic Control of Ca^2+^ Signaling in SCs and Neurite Outgrowth

**Figure 3:**
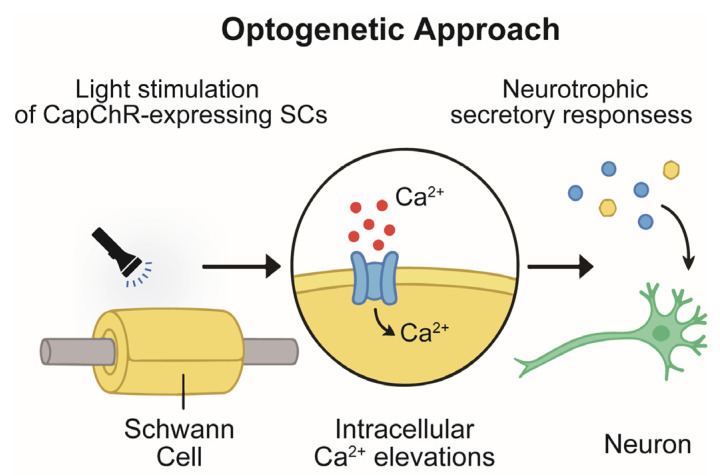
Optogenetic activation of CapChR2 in SCs induces Ca^2+^ influx and neurotrophic factor release that supports axonal regeneration. The blue and yellow circles indicate various types of neurotrophic factors conceptually represented as secreted molecules.

Optogenetics provides a powerful means to interrogate cellular signaling with high precision by using light-activated ion channels, most notably channelrhodopsins (ChRs). Originally developed for neuronal research, these microbial opsins act as light-gated cation channels that, upon photoactivation, permit the influx of monovalent and divalent cations. While traditionally applied to control membrane excitability in neurons, recent advances have expanded their application to glial biology, including SCs, where Ca^2+^ signaling plays a central role in repair phenotype induction and neurotrophic support. Among the engineered variants, Ca^2+^-permeable ChRs such as CapChR2 represent a particularly valuable tool for glial research. By enabling light-controlled Ca^2+^ influx with high spatiotemporal resolution, these actuators provide a selective approach to dissect Ca^2+^-dependent pathways in SCs. This complements conventional strategies—such as electrical stimulation or pharmacological modulation—by offering cell-type specificity and the ability to fine-tune Ca^2+^ dynamics without broadly affecting surrounding tissues. To facilitate accessibility for readers less familiar with this approach, Figure 3 summarizes the conceptual framework: light stimulation of CapChR2-expressing SCs evokes controlled Ca^2+^ entry, which subsequently triggers the release of neurotrophic and extracellular matrix components. These secretory responses act in an autocrine and paracrine manner to influence axonal survival, guidance, and regeneration, thereby highlighting how optogenetics can be used not only as an experimental tool but also as a conceptual bridge between ionic signaling and regenerative outcomes.

Classical channelrhodopsin-2 (ChR2), originally derived from Chlamydomonas reinhardtii, exhibits robust Na^+^ conductance but only limited Ca^2+^ permeability, with a PCa^2+^/PNa^+^ ratio typically less than 0.2 [67]. This limited divalent ion permeability constrains its applicability in studies aiming to activate Ca^2+^-dependent intracellular signaling pathways. In response, a series of engineered ChR2 variants—such as CatCh (Ca^2+^-translocating channelrhodopsin) [67], ChR2 (E123T/T159C) [68], and more recently, CapChR2—have been developed to enhance Ca^2+^ permeability while minimizing proton or sodium leakage [69].

CapChR2 is distinguished by its sustained Ca^2+^ conductance and time-dependent selectivity dynamics. In our patch-clamp recordings of CapChR2-expressing SCs, the initial PCa^2+^/PNa^+^ ratio reached approximately 1.2 within 10 s of light exposure, but decreased to ~0.7 by 30 s [70]. This temporal modulation suggests dynamic rearrangement of the channel pore or feedback regulation by intracellular signaling components.

At the structural level, crystallographic and computational studies implicate a cluster of acidic residues—E83, E90, D253 (collectively referred to as the “E-E-D triad”)—that are responsible for divalent cation selectivity [71,72,73]. Additional point mutations, such as L132C, S63D, and N238E, introduced through rational design, can further enhance Ca^2+^ selectivity by stabilizing hydrated or partially dehydrated Ca^2+^ at the selectivity filter [73]. Molecular dynamics simulations support the formation of transient coordination sites facilitating Ca^2+^ permeation even under low extracellular Ca^2+^ or negative membrane potential conditions.

A notable functional property of CapChR2 is its irradiance-dependent gating. At low light intensities (0.5–1 mW/mm^2^), CapChR2 can evoke sufficient intracellular Ca^2+^ elevations to activate downstream signaling without phototoxicity, while higher intensities (5–10 mW/mm^2^) produce more pronounced and sustained Ca^2+^ responses [72]. Unlike conventional ChR2, CapChR2 shows minimal desensitization under prolonged illumination, enabling chronic activation protocols—such as daily low-dose stimulation (e.g., 0.5 mW for 30 min; 900–1200 mJ/cm^2^)—suitable for regenerative applications in glial cells.

Our recent electrophysiological analysis further demonstrated that the half-maximal effective irradiance (EC_50_) of light-induced current for CapChR2 is approximately 0.76 mW/cm^2^ [70], suggesting that even low-intensity stimulation can reliably activate Ca^2+^ influx within a physiological range. Moreover, a residual Ca^2+^-permeable current persists transiently after cessation of illumination, providing an extended activation window that may contribute to prolonged intracellular signaling—an advantageous property for sustaining physiological effects such as neurotrophic factor release and neurite outgrowth.

These electrophysiological and structural characteristics translate into distinct functional outcomes. The transient high Ca^2+^ influx during the initial gating phase and sustained moderate Ca^2+^ entry thereafter are sufficient to activate canonical SC signaling pathways including ERK/MAPK, PI3K/AKT/PTEN, and NFAT [56,74]. Consistently, optogenetic stimulation of CapChR2-expressing SCs significantly enhanced neurite initiation and elongation in co-cultured PC12 cells, whereas vector-transfected or non-stimulated controls showed no such effects [70]. These results underscore that intracellular Ca^2+^ signaling, rather than passive membrane depolarization alone, governs the neurotrophic capacity of SCs.

**Figure 4:**
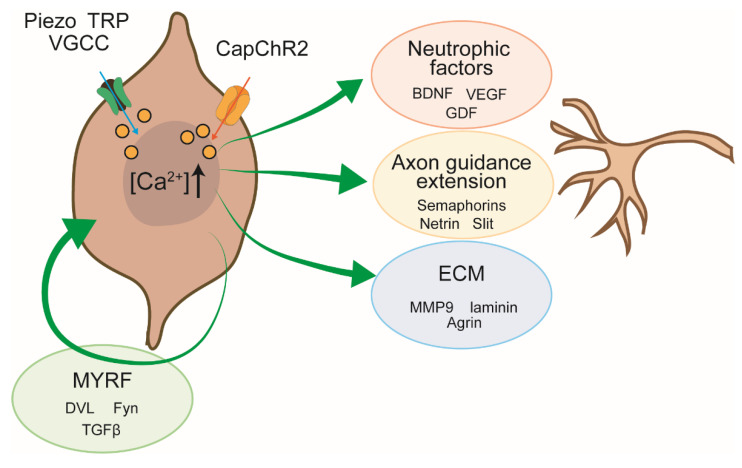
Neurite outgrowth pathways regulated by Ca^2+^ in SCs. TRP: transient re-ceptor potential; Piezo: mechanosensitive Piezo channels; VGCC: voltage-gated ion channels; CapChR2: a light-sensitive Ca^2+^-permeable channelrhodopsin; MYRF: myelin regulatory factor; ECM: extracellular matrix components.

To further elucidate the molecular basis of this neuritogenic effect, we conducted mass spectrometry-based proteomic profiling of the SC secretome under conditions of elevated intracellular Ca^2+^ influx. In our previous study utilizing Piezo2-mediated Ca^2+^ entry, we identified a wide range of secreted factors whose abundance was Ca^2+^-dependent [17]. Notably, this analysis revealed increased secretion of neurotrophic factors (e.g., brain-derived neurotrophic factor (BDNF), vascular endothelial growth factor (VEGF)), extracellular matrix (ECM) components (e.g., laminin subunits, heparan sulfate proteoglycans), and axon guidance molecules. These secreted elements are well documented to activate key axonal signaling pathways, including the PI3K/AKT and NFAT pathways, thereby promoting neurite extension and axonal guidance. The classification and functional categories of these Ca^2+^-induced secretory components are summarized in Figure 4 and Table 1.

Schematic summary of Ca^2+^-dependent signaling pathways in SCs that promote neurite outgrowth during peripheral nerve regeneration. Elevation of intracellular Ca^2+^—via endogenous ion channels (e.g., Piezo, TRP, VGCC) or optogenetic actuators (e.g., CapChR2)—activates multiple downstream pathways that converge on neuro-supportive outputs. Key Ca^2+^-regulated outputs include: Neurotrophic factors, Axon guidance cues, ECM components, MYRF. These Ca^2+^-induced pathways operate in both autocrine and paracrine fashions to create a permissive microenvironment for axonal regrowth.

Taken together, these data suggest that CapChR2 functions as a powerful optogenetic tool for modulating SC behavior in a light-dependent, Ca^2+^-specific manner. Its precise biophysical properties—including high Ca^2+^ selectivity, illumination-dependent gating, and resistance to desensitization—enable the fine-tuned control of SC-derived neurotrophic support. This technology holds significant promise for both fundamental studies of glial-axon communication and the development of regenerative strategies targeting peripheral nerve injury and demyelinating diseases.

## 5. Calcium-Triggered Secretory Programs in Schwann Cells: Implications for Nerve Regeneration

Optogenetic activation of SCs via CapChR2 provides a cell-type–targetable approach to modulate glial function in peripheral nerve regeneration. Through light-induced depolarization and subsequent Ca^2+^ influx, optogenetics allows precise temporal and spatial control of intracellular signaling pathways important for SC plasticity and regenerative phenotype. Similar enhancements in neuro-supportive behavior have also been supported following electrical stimulation, which increases NGF secretion and neurite outgrowth by modulating SC phenotype [139]. This suggests that both optical and electrical biophysical cues can reprogram SC function to favor regeneration. This technique offers distinct advantages over traditional stimulation strategies, especially in its cell type-specific modulation capabilities and minimal off-target effects [140,141]. These insights are further supported by in vivo evidence showing that SC transplantation facilitates remyelination, functional recovery, and tissue sparing in spinal cord injury models, reinforcing the therapeutic promise of SC-based interventions beyond the peripheral nervous system [142]. While the predominant influence of SC Ca^2+^ elevations on neuronal regeneration are mediated by the paracrine release of neurotrophic factors, extracellular matrix proteins, and axon guidance cues, potential direct effects at the axon–glia interface should also be considered. Localized Ca^2+^ signaling in periaxonal SC domains may regulate adhesion molecules and ATP release, thereby modulating axonal excitability and growth cone behavior. In addition, specialized structures such as Schmidt–Lanterman incisures and paranodal loops provide potential routes for ionic and metabolic coupling, suggesting that SC Ca^2+^ dynamics may exert both indirect and direct influences on neuronal machinery during regeneration.

Mechanistically, Ca^2+^ influx—whether endogenous (e.g., Piezo/TRP/SOCE/VGCCs) or optogenetic (CapChR2-mediated)—activates multiple canonical pathways, including PI3K/AKT, calcineurin/NFAT, and MAPK/ERK. These cascades converge into transcriptional and translational programs that drive the expression and secretion of a wide range of bioactive molecules. The resulting secretome plays a central role in orchestrating peripheral nerve repair, acting on both neurons and glia via paracrine and autocrine mechanisms (see Figure 4). Here, we discuss the effects of these factors on neurons and SCs based on our Ca^2+^-triggered secretome dataset obtained via Piezo2-mediated entry (Table 1; ref. [17]).

### 5.1. Axon Guidance

Netrin/Slit/ROBO module: Mediates chemoattractive and chemorepulsive gradients essential for initial axonal pathfinding.

Among the guidance molecules, netrin-1, Slit1, and their receptors (e.g., DCC, Unc5, ROBO1) constitute a functionally coherent group central to axon pathfinding. Netrin-1, originally identified as a chemoattractant secreted by floor plate cells, directs commissural axons in the developing spinal cord through interactions with DCC and integrins [75,76]. Its activity is context-dependent, eliciting attraction or repulsion based on receptor composition and intracellular signaling, such as FAK and Src family kinase activation [76]. Netrin signaling has also been implicated in cortical neuron migration and psychiatric disorders [77].

Slit proteins, functioning primarily as chemorepellents via ROBO receptors, cooperate with netrin-1 in establishing complex axonal trajectories. In the thalamocortical system, overlapping gradients of Slit1 and netrin-1 generate emergent axonal responses not elicited by either cue alone [78]. Notably, subthreshold concentrations of Slit1 have been shown to facilitate netrin-1–mediated attraction, underscoring the synergistic and combinatorial nature of axon guidance signaling [79]. The Slit/ROBO axis is further modulated by miRNAs such as miR-92, which regulates Robo1 translation, thereby tuning commissural axon sensitivity to repulsive cues and enabling midline crossing [85,86]. These mechanisms are supported by broader insights into Slit/ROBO signaling, which is now recognized to operate not only in neuronal systems but also in diverse cell types, including leukocytes and epithelial cells, where it governs processes such as chemotaxis and barrier formation [87].

Collectively, these findings highlight the capacity of Ca^2+^-activated SCs to secrete a spectrum of axon guidance cues—netrin-1, Slit1, ROBO1—that operate in a spatially and temporally orchestrated fashion. Their coordinated action likely contributes to directed neurite extension, axonal sorting, and reinnervation, suggesting that the netrin/Slit/ROBO module functions as a conserved, Ca^2+^-sensitive regulatory node in peripheral nerve repair.

Semaphorin/Plexin/Neuropilin axis: Functions predominantly as repulsive guidance signaling to ensure spatial fidelity of regrowth.

In parallel, the Semaphorin–Plexin/Neuropilin axis represents another major regulatory module of axonal navigation and synaptic patterning. Semaphorins, including secreted class III members (e.g., Sema3A, Sema3F) and membrane-bound forms (e.g., Sema6A), act predominantly as repulsive cues that modulate growth cone dynamics during regeneration [81,83,84]. These ligands are interpreted by receptor complexes composed of Plexin-A family members and co-receptors such as Neuropilin-2 [82,88,89,93,94]. SC-derived semaphorin signals, released following Ca^2+^ influx via CapChR2 or Piezo2, likely function to constrain inappropriate axonal trajectories, ensuring spatial fidelity of reinnervation and preventing circuit miswiring.

Semaphorin signaling is not only crucial during development but also restricts regenerative axon growth, as shown in C. elegans models where deletion of plexin genes leads to increased regrowth and reconnection of motor neuron neurites [84]. Furthermore, recent studies underscore the dynamic regulation of synaptogenesis, excitatory-inhibitory balance, and electrical activity-dependent refinement by semaphorin pathways [83]. Neuropilin-2, in particular, mediates selective responses to Sema3F and is essential for proper development of cranial nerves and sensory axon projections in mammals, indicating its conserved role in axonal guidance [93,94]. Plexin-A4, a key component of these signaling complexes, mediates responses to both secreted (e.g., Sema3A) and membrane-bound (e.g., Sema6A) semaphorins and contributes to the guidance of peripheral sensory and sympathetic ganglion axons, as well as central projections such as the anterior commissure [88]. Supporting this, Leighton et al. (2001) demonstrated the axon guidance function of Sema6A and the importance of Plexin-A signaling in brain wiring through gene-trap strategies in mice [89]. Thus, the coordinated action of Semaphorin–Plexin/Neuropilin signaling serves to shape precise regenerative outcomes during SC-mediated peripheral nerve repair.

Neuropilin-2 serves as a critical receptor component for Sema3F-mediated signaling and plays an indispensable role in directing axonal projections during neural development. Experimental analyses using Npn-2 knockout mice have revealed that the absence of Neuropilin-2 leads to disorganized formation of several cranial and spinal sensory nerves, along with aberrant axonal trajectories in the anterior commissure and hippocampal mossy fibers [93,94]. These findings underscore the selectivity of Neuropilin-2 for Sema3F, distinguishing it functionally from Neuropilin-1, which preferentially mediates responses to Sema3A. The observed deficits in Npn-2-deficient mice highlight its context-dependent function in both CNS and PNS axon guidance, suggesting that Neuropilin-2 may play a similarly precise role during peripheral nerve regeneration, particularly when released in a Ca^2+^-dependent manner from activated SCs.

Ephrin/Eph system: Provides fine-tuned, contact-mediated signaling for topographical axonal targeting and synapse formation.

Ephrin–Eph signaling represents yet another critical layer of contact-mediated guidance that functions through bidirectional signaling between membrane-bound Eph receptor tyrosine kinases and their ligands. EphA2, one of the Eph receptors, transduces both repulsive and attractive cues via cell–cell interactions and plays a central role in modulating growth cone behavior through reorganization of the actin cytoskeleton [90,91,92]. These effects are mediated via downstream activation of Rho family GTPases, including RhoA, Rac1, and Cdc42. Notably, Eph–ephrin signaling can operate in either a binary or proportional fashion, enabling context-dependent axonal discrimination within complex topographical maps [92]. The presence of EphA2 or its ligands in the Ca^2+^-induced secretome of SCs suggests a mechanism by which axon pathfinding is locally regulated with high spatial precision. Bidirectional signaling, together with ectodomain cleavage and endocytosis of Eph/ephrin complexes, further refines axonal responsiveness during regeneration, indicating a high level of plasticity in EphA2-dependent guidance programs.

SCs activated by Ca^2+^ signals function not simply as supporting cells but as an autonomous and precise source of axon guidance factors. In particular, the cooperative action of Netrin-1 and Slit1 and the pathway restriction by the Semaphorin-Plexin axis are important for achieving accurate regeneration and functional reconnection. Future work should resolve how timing, localization, and concentration-dependence of these cues intersect with regeneration outcomes.

### 5.2. Neurotrophic Factors

In parallel with axon guidance cues, Ca^2+^ influx robustly enhances the expression and secretion of multiple neurotrophic factors from SCs, including brain-derived neurotrophic factor (BDNF), vascular endothelial growth factor A (VEGF-A), stromal cell-derived factor-1 (CXCL12/SDF-1), and growth differentiation factor 7 (GDF7). In addition to their endogenous secretory capacity, SCs have been shown to enhance the viability and neuronal differentiation of co-cultured neural stem cells via secretion of BDNF and GDNF, reinforcing their neurotrophic roles in both developmental and regenerative contexts [143]. Collectively, these molecules support neuronal survival, axonal elongation, synaptogenesis, and remodeling of the neural milieu [18,95,96,97,98,99,100,101,102,103,104,105,106,107].

BDNF, acting through TrkB and p75 (NTR) receptors, orchestrates a broad range of functions in neurons, from activity-dependent synaptic refinement to structural plasticity. It is involved in both central and peripheral nervous system development and repair, with downstream activation of CREB-, MAPK-, and PI3K-dependent pathways [99,101,102]. BDNF levels also correlate with improved neuronal resilience under stress and have been implicated in the modulation of metabolism and psychiatric disease pathophysiology.

VEGF-A, though classically known for its angiogenic function, exerts direct neurotrophic and neuroprotective actions by binding to VEGFR2/Flk-1 receptors expressed on neurons and glia. It enhances neurogenesis, axonal guidance, and neuronal survival both in vitro and in vivo [95,96,97,98]. Thus, SC-derived VEGF-A may simultaneously promote revascularization and axonal repair, contributing to coordinated neurovascular regeneration.

CXCL12 (SDF-1), a chemokine enriched in the perivascular niche after injury, binds to its receptor CXCR4 to guide neuronal precursor migration and axon pathfinding. In mature neurons, it supports neurite outgrowth and confers resistance to inhibitory molecules in the glial scar, via activation of the PI3K/AKT/mTOR pathway [105,106,107]. This dual role in development and repair highlights its utility in orchestrating context-dependent responses to injury.

GDF7, a member of the bone morphogenetic protein (BMP) family, is selectively expressed in the dorsal roof plate and is essential for specifying subsets of commissural interneurons in the spinal cord [104]. Its detection in our Ca^2+^-triggered secretome dataset (Table 1) suggests reactivation of developmental programs in adult SCs, potentially aiding restoration of discrete circuitry.

Collectively, the Ca^2+^-induced secretome from SCs provides a dynamic trophic scaffold that supports both neuronal and glial populations during regeneration. These neurotrophic factors likely operate in both paracrine and autocrine manners to sustain the repair phenotype of SCs, synergize with contact-mediated guidance cues, and optimize the spatial and temporal fidelity of axonal reinnervation.

Beyond their neural targets, SC-derived neurotrophic factors and neuromodulators may also influence adjacent non-neuronal cells, including keratinocytes and dermal fibroblasts, suggesting broader roles in peripheral tissue homeostasis. This emerging perspective aligns with recent concepts from the field of neurocosmetics, which propose that peripheral nerves—through the activity of SCs—modulate skin physiology, barrier integrity, and even emotional responses via the skin–brain axis [144,145,146,147]. The skin is richly innervated and expresses receptors for neuromediators such as β-endorphins, serotonin, and substance P [148], all of which may be influenced by Ca^2+^-activated SCs. These findings suggest that targeted activation of SCs could not only promote neural regeneration but also enhance cutaneous resilience and psychophysiological well-being, as recently discussed in the context of neurocutaneous regulation and sensory-responsive skincare [148].

### 5.3. Extracellular Matrix (ECM) Molecules

In addition to soluble neurotrophic factors and axon guidance cues, SCs activated by Ca^2+^ influx secrete a diverse repertoire of extracellular matrix (ECM) molecules that create a structurally supportive and bioactive microenvironment conducive to regeneration. These ECM components not only provide mechanical scaffolding for axonal outgrowth but also act as modulators of cell signaling, adhesion, and cytoskeletal dynamics. Importantly, the ECM landscape sculpted by Ca^2+^-activated SCs is increasingly recognized as a critical determinant not only for peripheral nerve regeneration but also for broader influences on neural circuit remodeling, including in autonomic and central nervous system contexts.

Among structural and adhesive glycoproteins, laminin β2 constitutes a foundational component of the SC basal lamina; fibronectin forms an injury-associated provisional matrix; type IX collagen (COL9A1) contributes to specialized matrices such as perineuronal nets. Laminin β2 acts via integrin and dystroglycan receptors to promote myelination and directed growth cone extension [149,150], while fibronectin mediates SC adhesion and migration in a Ca^2+^-dependent manner and supports early neurite sprouting [151,152].

Collagen IX (COL9A1), though less abundant, plays a critical role in synaptic organization and perineuronal net (PNN) maintenance. Its loss leads to disassembly of PNNs and aberrant inhibitory synapse formation in the telencephalon, suggesting roles in both peripheral and central neuroplasticity [118,119].

Matrix components involved in synaptic and nodal specialization further illustrate the multifaceted influence of SCs beyond structural support. Agrin, a heparan sulfate proteoglycan, is indispensable for the formation and stabilization of the neuromuscular junction (NMJ), where it orchestrates postsynaptic differentiation and acetylcholine receptor clustering [108,153]. In addition to its canonical role at the NMJ, agrin also regulates synaptogenesis in central circuits, including hippocampal neurons, by controlling vesicular turnover and excitatory synaptic differentiation [110]. Recent studies show that agrin modulates neuronal responsiveness to excitatory neurotransmitters and Ca^2+^-mediated signaling, indicating broader functions in CNS excitability and injury responses [109]. Its precise deposition at presynaptic terminals is facilitated by MMP14-mediated ECM remodeling, further linking agrin function to dynamic matrix turnover [108].

Osteopontin (OPN), a multifunctional glycoprotein, is increasingly recognized as a critical modulator of inflammatory and regenerative signaling within the nervous system. OPN is secreted by both neurons and glia under pathological conditions and is upregulated in response to injury and neuroinflammation [114,154]. It interacts with integrins (e.g., αvβ3) and CD44 to orchestrate astrocyte migration, microglial activation, and extracellular matrix remodeling. In ALS models, OPN expression correlates with selective motor neuron vulnerability and induces matrix metalloproteinase-9 (MMP9) expression in a subtype-specific manner, implicating it in delayed-phase neurodegeneration and compensatory plasticity [113]. In frontotemporal dementia (FTD), elevated OPN levels in MAPT-mutant neurons are associated with impaired graft survival and reactive gliosis, whereas knockdown of OPN improves integration and reduces inflammatory responses [114]. These findings collectively underscore osteopontin’s dual role as an immune modulator and matrix effector, coupling inflammatory cascades with cytoskeletal remodeling during nerve regeneration.

Through the activity-dependent secretion of agrin and osteopontin, SCs participate in the re-establishment of synaptic architecture and functional connectivity. These molecules not only maintain nodal integrity and synaptic fidelity in the peripheral nervous system but also influence neuroimmune dynamics and synaptic plasticity in the central and autonomic nervous systems, reinforcing their relevance to global neural homeostasis and regeneration.

Heparan sulfate proteoglycan glypican-1 and cytoskeletal linker MACF1 further support SC-mediated regeneration. Glypican-1 modulates the responsiveness of SCs and neurons to neurotrophic factors including BDNF, VEGF, and neuregulin through its interactions with heparan sulfate-binding domains and signaling receptors. It also participates in the differentiation of neuron-like cells via complex formation with neuregulin and contributes to SC myelination through its interaction with collagen α4(V) [120,121,122]. MACF1 (microtubule-actin crosslinking factor 1) links cytoskeletal structures to ECM anchors, promoting SC polarity and migration across the regenerating nerve bridge [124].

Finally, MMP9, a matrix metalloproteinase, plays a multifaceted role in sculpting the regenerative niche. In addition to degrading structural ECM proteins, MMP9 liberates matrix-sequestered growth factors, modulates laminin-integrin signaling, and facilitates neuronal survival through PI3K/AKT pathway activation [115,117]. MMP9 activity is tightly regulated during critical developmental windows and injury responses, and its dysregulation is associated with impaired neuroplasticity and neuropsychiatric conditions [116]. Because endoneurial fibroblasts contribute substantially to peripheral nerve ECM, careful cell-type purification is required to attribute MMP9-dependent remodeling specifically to SCs; MACS-based purification and transcriptomic benchmarking provide practical standards [155].

Together, these ECM molecules comprise a finely tuned secretome that is activated in response to Ca^2+^ entry, acting in synergy with neurotrophic factors to define the spatial architecture and biochemical gradient fields necessary for guided axonal regrowth. Beyond peripheral nerve repair, such Ca^2+^-induced ECM remodeling may also impact the reorganization of autonomic circuits and even exert translatable effects in the central nervous system via long-range SC signaling or ECM-derived vesicular transport. Thus, the optogenetically activated ECM profile not only restores structural continuity but also lays the molecular groundwork for functional reintegration across peripheral and central domains of the nervous system. Furthermore, the regenerative potential of SC-derived ECM has been harnessed in biomaterial-based transplantation strategies, where SCs or their secreted matrices are embedded in engineered scaffolds to mimic the native niche and promote repair [156].

### 5.4. SC-Autonomous Regulation via MYRF and Associated Intracellular Signaling Cascades

Beyond providing structural and paracrine support to neurons, SCs possess robust intrinsic mechanisms that regulate their own regenerative and myelinating phenotypes. A key emerging factor in this context is the myelin regulatory factor (MYRF), a membrane-associated transcription factor originally characterized in oligodendrocyte differentiation. MYRF has recently been implicated in peripheral nerve regeneration, where it supports transcriptional reprogramming in repair SCs.

Upon proteolytic cleavage, MYRF translocates to the nucleus and promotes the expression of myelin-related genes such as Mbp and Prx, thereby maintaining glial identity under regenerative conditions. In peripheral nerves, MYRF expression is upregulated after injury and functions in concert with canonical SC transcription factors including SOX10, EGR2 (Krox20), and c-Jun, establishing a dynamic regulatory network that integrates injury cues, intracellular Ca^2+^ signaling, and epigenetic modulation [129,130].

Importantly, intracellular Ca^2+^ influx, triggered by optogenetic or endogenous channel activation, may serve as a proximal trigger for MYRF activation, positioning it as a key effector in Ca^2+^-dependent regenerative cascades. This highlights the dual role of SCs—not only as providers of neurotrophic support but also as autonomous regulators of their own phenotype via Ca^2+^-driven transcriptional programs [126].

MYRF-dependent transcriptional activity extends beyond myelin gene expression. It also regulates genes involved in lipid biosynthesis (e.g., Fasn, Srebp1), mitochondrial function (e.g., Pgc1α, Mfn2), and autocrine factors such as neuregulin-1 (NRG1) and insulin-like growth factor-1 (IGF-1), all of which support SC polarity, metabolic fitness, and remyelination. In particular, NRG1 engages ErbB receptors in an autocrine feedback loop, reinforcing a pro-regenerative phenotype [134], while IGF-1 supports cytoskeletal reorganization and mitochondrial homeostasis.

Additional regulatory input is provided by non-canonical Wnt signaling, notably through Wnt5a–DVL1. These pathways are activated in SCs following nerve injury and contribute to cytoskeletal remodeling, polarity, and directed migration [125,126]. Wnt5a is expressed in both human and rodent injured nerves and, via DVL1 and Fyn kinase, influences SC dynamics and axon guidance. Fyn itself, a Src-family tyrosine kinase, is a critical mediator of SC differentiation and myelination and operates downstream of multiple receptor systems [133].

GSK3β represents another intracellular node coordinating SC behavior. It is a key target of the PI3K pathway, and its inhibition leads to β-catenin accumulation and transcription of genes promoting SC proliferation and differentiation [135,136]. Activation of the GSK3β/β-catenin pathway, as demonstrated in SCs treated with low-intensity pulsed ultrasound or Wnt agonists, enhances viability, proliferation, and neurotrophin expression [132,136].

TGF-β1 is another major modulator of SC fate. It regulates gene expression at the transcriptional level, suppressing myelination-associated factors while enhancing non-myelinating and migratory phenotypes through activation of SMAD2/3 and MAPK/NF-κB pathways [127,129,131]. TGF-β1 also induces expression of neurotrophic cytokines such as leukemia inhibitory factor (LIF) [128], which play roles in supporting neuronal regeneration. Interestingly, TGF-β1-mediated c-Jun activation can lead to SC apoptosis in specific developmental contexts, suggesting its role as both a differentiation and survival regulator [130].

On the other hand, PTEN serves as a critical gatekeeper of SC plasticity. By antagonizing PI3K/AKT signaling, PTEN restricts uncontrolled SC proliferation and maintains differentiation balance. Loss of PTEN enhances regenerative responses but may predispose SCs to malignant transformation, as observed in models of malignant peripheral nerve sheath tumors (MPNSTs) [137]. Moreover, PTEN dysfunction disrupts neuromuscular junction development by impairing Agrin signaling and autophagy, emphasizing its role in both regeneration and developmental integrity [138].

In summary, MYRF operates at the convergence of intracellular Ca^2+^ signaling and transcriptional control, functioning as a key node within an autocrine regulatory circuit that maintains SC regenerative competence. Through coordinated activation of signaling molecules such as Wnt5a, TGF-β1, Fyn, GSK3β, and PTEN, SCs initiate and sustain cell-intrinsic programs that integrate environmental cues, metabolic demands, and structural remodeling to drive successful peripheral nerve regeneration.

Taken together, these findings underscore the notion that SCs, when appropriately stimulated via Ca^2+^ influx mechanisms such as Piezo2 or optogenetic tools, act as central coordinators of peripheral nerve regeneration. Their secretory activity supports both neuronal repair and glial remodeling, making them an attractive target for therapeutic neuromodulation strategies. From a translational perspective, several therapeutic modalities aim to mitigate the consequences of peripheral nerve injury and promote regeneration by engaging or complementing Ca^2+^-dependent SC secretory programs. These include autologous or processed nerve grafts that preserve native trophic cues [157,158], electrical stimulation protocols that enhance activity-dependent Ca^2+^ influx and neurotrophic factor secretion [159,160,161], pharmacological modulators targeting TRP, Piezo, or purinergic receptors [162,163,164], and emerging cell- or exosome-based therapies that provide trophic and immunomodulatory support [165,166]. While the present review has focused on the mechanistic basis of Ca^2+^ signaling and secretion, acknowledging these approaches underscores the translational potential of targeting SC secretory pathways in regenerative medicine.

## 6. Conclusions

Recent advances in optogenetics have provided powerful tools to dissect and manipulate glial cell function with unprecedented spatial and temporal resolution. Among these, CapChR2 stands out as a rationally engineered optogenetic actuator that enables sustained, Ca^2+^-permeable (Ca^2+^-biased) influx in SCs, helping to circumvent limitations of classical channelrhodopsins. By precisely modulating intracellular Ca^2+^ dynamics, CapChR2 activation orchestrates a cascade of regenerative processes, including enhanced secretion of neurotrophic factors, reprogramming toward repair phenotypes, and support of neurite outgrowth in co-cultured neurons.

Our findings, supported by electrophysiological analyses and proteomic profiling, demonstrate that light-induced SC activation via CapChR2 can recapitulate key features of physiological Ca^2+^ signaling and achieve functionally meaningful outcomes for axonal regeneration. Compared with electrical or mechanical stimulation, this strategy offers unique advantages in cell-type-targetable specificity, fine spatiotemporal control, and compatibility with chronic, low-intensity stimulation protocols.

Nonetheless, realizing its full therapeutic potential will require addressing outstanding challenges in gene delivery, light penetration/dosing (phototoxicity and thermal load), and biocompatibility/immune responses in vivo. Integration with novel photonic systems, tissue-specific promoters, and clinically viable gene transfer vectors will be crucial steps toward translational application.

In summary, CapChR2-mediated optogenetic control of SCs offers the following: (1) precise modulation of intracellular Ca^2+^ signaling; (2) enhancement of neurotrophic and regenerative secretory profiles; and (3) cell-type-targetable, minimally perturbative intervention potential. These features highlight its promise not only for dissecting SC biology but also for enabling targeted interventions in peripheral nerve and demyelinating disorders—especially when combined with complementary approaches such as bioengineered scaffolds and controlled electrical/biophysical conditioning. Looking forward, future research should integrate multi-omics profiling to comprehensively map Ca^2+^-triggered secretory networks, advance bioelectronic and optogenetic tools for precise SC modulation, and evaluate their therapeutic potential in preclinical models of chronic or large-gap nerve injuries.

## Figures and Tables

**Table 1:** Classification of SC-derived secretory factors and their functional category (from [17]). Colors indicate correspondence with the function categories shown in Figure 4.

Protein Name	Gene Symbol	Function Category	References
Netrin-1	NTN1	Axon Guidance	[75,76,77]
Slit1	SLIT1	Axon Guidance	[78,79,80]
Semaphorin	SEMA	Axon Guidance	[81,82,83,84]
ROBO1	ROBO1	Axon Guidance	[85,86,87]
Plexin-A4	PLXNA4	Axon Guidance	[88,89]
EphA2	EPHA2	Axon Guidance	[90,91,92]
Neuropilin-2	NRP2	Axon Guidance	[93,94]
VEGF-A	VEGFA	Neurotrophic Factor	[95,96,97,98]
BDNF	BDNF	Neurotrophic Factor	[99,100,101,102]
GDF7	GDF7	Neurotrophic Factor	[103,104]
CXCL12	CXCL12	Neurotrophic Factor	[105,106,107]
Agrin	AGRN	ECM	[108,109,110]
Laminin β2	LAMB2	ECM	[111,112]
Osteopontin	OPN	ECM	[113,114]
MMP9	MMP9	ECM	[115,116,117]
COL9A1	COL9A1	ECM	[118,119]
Glypican-1	GPC1	ECM	[120,121,122]
MACF1	MACF1	ECM	[123,124]
Wnt5a	WNT5A	SC Regulation	[125,126]
TGFβ1	TGFB1	SC Regulation	[127,128,129,130,131]
DVL1	DVL1	SC Regulation	[126,132]
Fyn	FYN	SC Regulation	[133,134]
GSK3B	GSK3B	SC Regulation	[135,136]
PTEN	PTEN	SC Regulation	[125,137,138]

## Abbreviations

The following abbreviations are used in this manuscript:

AKT	Protein kinase B
AGRIN	Agrin
BDNF	Brain-derived neurotrophic factor
Ca^2+^	Calcium ion
CapChR2	Calcium-permeable Channelrhodopsin-2
COL9A1	Collagen type IX alpha 1 chain
CXCL12 (SDF-1)	Stromal cell-derived factor-1
DVL1	Disheveled segment polarity protein 1
ECM	Extracellular matrix
EPHA2	Eph receptor A2
ER	Endoplasmic reticulum
ERK	Extracellular signal-regulated kinase
GDF7	Growth differentiation factor 7
GSK3β	Glycogen synthase kinase 3 beta
LIF	Leukemia inhibitory factor
MAPK	Mitogen-activated protein kinase
MACF1	Microtubule-actin crosslinking factor 1
MMP9	Matrix metalloproteinase 9
MYRF	Myelin regulatory factor
NFAT	Nuclear factor of activated T cells
NMJ	Neuromuscular junction
NRP2	Neuropilin-2
NTN1	Netrin-1
OPN	Osteopontin
PI3K	Phosphoinositide 3-kinase
PLXNA4	Plexin-A4
PNN	Perineuronal net
PTEN	Phosphatase and tensin homolog
ROBO1	Roundabout guidance receptor 1
SCs	Schwann cells
SEMA	Semaphorin
SOCE	Store-operated calcium entry
TGF-β1	Transforming growth factor beta 1
TRP	Transient receptor potential
VGCC	Voltage-gated calcium channel
VEGF-A	Vascular endothelial growth factor A
WNT5A	Wnt family member 5A
